# Preparation of Montmorillonite–Melamine Cyanurate and Inhibition of the Emission of Phosphine from PA6/Aluminum Hypophosphate

**DOI:** 10.3390/polym16202946

**Published:** 2024-10-21

**Authors:** Lin Wang, Yuyang Li, Chenyang Yan, Xiangmei Li, Jiyu He, Rongjie Yang

**Affiliations:** National Enginneering Research Center for Flame Retardant Materials, School of Materials Science & Engineering, Beijing Institute of Technology, 5 Zhongguancun South Street, Haidian District, Beijing 100081, China; 3120235237@bit.edu.cn (L.W.); 3120211151@bit.edu.cn (Y.L.); 3220212088@bit.edu.cn (C.Y.); hejiyu@bit.edu.cn (J.H.); yrj@bit.edu.cn (R.Y.)

**Keywords:** intercalation, montmorillonite, aluminum hypophosphite, phosphine, polyamide 6

## Abstract

In order to mitigate the release of toxic phosphine from aluminum hypophosphite in twin-screw processing, montmorillonite–melamine cyanurate was prepared by three methods: (1) mechanical intercalation, (2) water intercalation and (3) in situ intercalation. The sheet spacing of montmorillonite was increased from 1.140 nm to 1.141 nm, 1.208 nm and 1.217 nm for these three methods, respectively, and scanning electron microscope (SEM) and transmission electron microscopy (TEM) proved that melamine cyanurate was successfully inserted into the montmorillonite sheets. The montmorillonite–melamine cyanurate from in situ intercalation can best inhibit the release of PH_3_ from aluminum hypophosphite, and the peaks of phosphine, mean values of phosphine and integral of phosphine were reduced by 81.9%, 72.1% and 72.2%, respectively. The mode of action of montmorillonite–melamine cyanuric inhibition of the emission of phosphine from aluminum hypophosphite can be attributed to the physical absorption of montmorillonite and the chemical bonding of melamine cyanurate. In addition, in situ intercalation can slightly improve flame retardancy, attributed to incomplete exfoliation of montmorillonite sheets.

## 1. Introduction

Polyamide 6 has excellent mechanical properties, wear resistance, chemical resistance and processing properties, so it is widely used in textile, automotive, electronic and electrical industries. However, polyamide 6 has poor flame retardancy, which limits its wider application [1,2,3]. In recent decades, halogen-free flame retardant containing phosphorus, nitrogen, silicon and metal oxides is attracting more and more attention in flame retardant research [4,5,6].

Aluminum hypophosphate (AHP), a kind of cheap and halogen-free inorganic phosphorus flame retardant, performs excellent flame retardancy, attributed to a 41.98 wt% high content of phosphorus. Therefore, aluminum hypophosphate is widely applied to flame retardant polymers [7,8,9,10,11,12]. However, AHP decomposes and releases phosphine gas (PH3) in the process of high-temperature processing of twin-screw extruder, because of its sensitivity to heat and shear [13]. PH3 gas can be accumulated and ignited easily during processing, and even explode in extreme cases [14]. In addition, PH3 gas can easily be inhaled into the lungs at a processing site or fire site, and causes unpredictable damage to the sympathetic nervous system, blood system and lung cells of people without respiratory protection [14]. Therefore, inhibiting the release of PH_3_ has very important practical significance for the promotion of the application of AHP.

To date, the release of PH_3_ from AHP can be inhibited by microencapsulation and synergistic compounding. Microcapsule technology, a very mature technology, has been widely used in various fields of research [15,16]. By forming a shell on the surface of AHP particles, microencapsulation technology gives special properties to the AHP, the core material, to enhance the application of AHP [17]. Ge [13] successfully microencapsulated AHP with melamine cyanurate by in situ polymerization. The results of TGA-FTIR showed that the amount of PH_3_ released was reduced and the release time was delayed. Compared with microcapsule technology, synergistic compounding is easier to be implemented with modification by simple processing [18]. Therefore, some substances absorbing or reacting with PH_3_ to form non-gaseous substances can be synergistically blended with AHP to inhibit the release of PH_3_. Yuyang Li [19] used metal–organic frameworks (MOFs) with large specific surface area to synergistically compound with AHP. PH_3_ release curves of both the processing process and TGA-FTIR showed that the PH_3_ release was successfully mitigated. There is little clear research on mitigating PH_3_ release of AHP except the above two mentioned studies [13,19].

Montmorillonite (MMT), an economical, available and environmentally friendly adsorbent, possesses distinctive and excellent attributes including unique two-dimensional special structure, mineral surface adsorption, interlayer cation exchange, pore filtration and special nanoscale structural effects [20,21,22,23]. Owing to the high special surface area and porous structure, MMT can effectively adsorb waste through various interactions, such as capillary forces, surface tension, hydrogen bonding and van der Waals forces [24,25]. In addition, MMT is also a good anti-drip agent [19]. Therefore, MMT is applied to flame retardancy [26], catalytic carrier [27], drug loading [28], wastewater treatment [29], attributed to excellent thermal stability, gas barrier ability [30,31], strong adhesion, and large specific area and adsorption ability [32]. MMT can be transformed into more efficient MMT-based adsorption materials through modification, activation or composite modification. The usual modification methods include activation, inorganic filling, organic intercalation, polymer intercalation and inorganic–organic composition [33]. Therefore, cheap and widely available MMT is a unique choice for a PH_3_ absorbent.

Melamine cyanurate (MCA) is a typical flame retardant for polyamides 6 and 66, attributed to high flame retardancy, non-toxic product, low smoke release and low price. The decomposition of MCA leads to the sublimation of melamine and the degradation of cyanuric acid [34,35,36]. The amino group in melamine can react with PH_3_ to form a non-gaseous compound; thereby, the release of phosphine gas can be inhibited [19]. Furthermore, Zhao Min [37] successfully achieved the preparation of montmorillonite–melamine cyanuric (MMT-MCA) by intercalation. Therefore, MMT-MCA can be prepared and applied as a better PH_3_ absorbent than raw MMT.

In this work, MMT-MCA was prepared by three methods: (1) in situ intercalation, (2) water intercalation and (3) mechanical intercalation. The three kinds of MMT-MCA compounded with AHP were applied to PA6, and the effect of the intercalation method and loading proportion of MMT-MCA on the emission of PH_3_ from AHP in the processing of twin-screw extrusion and flame retardant were studied. The results showed that in situ intercalation could best inhibit the emission of PH_3_ by 72% and slightly improve LOI and UL 94.

## 2. Materials and Methods

### 2.1. Materials

Sodium-montmorillonite (MMT) was produced by Nanocor Inc. (Chapel Hill, NC, USA). Melamine (MA), cyanuric acid (CA) and melamine cyanurate (MCA) were purchased from Aladdin (Shanghai) Co., LTD., Shanghai, China. Nylon 6 was purchased from Changle Hengshen Synthetic Fiber Technology Co., LTD., Fuzhou, China. Aluminum hypophosphate (AHP) was purchased from Hunan Meilaipo Technology Development Co., LTD., Yongzhou, China.

### 2.2. Preparation of MMT-MCA

(1) In situ intercalation

MMT−MCA was prepared by in situ intercalation according to the previous literature in our laboratory [37]. First, 49.5 g cyanuric acid (CA) and 50.5 g melamine (MA) were individually dissolved in 3.0 L deionized water in 95 °C under magnetic agitation for 2 h. Next, 100.0 g of MMT was dispersed in deionized water in a 50 L reactor with mechanical agitation and high–low-temperature circulator at 85 °C, and the temperature of the reactor increased to 95 °C two hours later. Then, CA hot water dispersion was slowly added to the reactor by a metering pump at a constant speed for about 30 min and the reaction continued to be stirred for 3 h. Then, MA hot water dispersion was slowly added to the reactor by a metering pump at a constant speed for about 30 min and the reaction continued to be stirred for 3 h. Finally, the product named 2# was obtained by centrifugation, drying and grinding.

(2) Water intercalation

First, 100.0 g MCA was dispersed in 5.0 L deionized water in 95 °C under magnetic agitation for 2 h. Next, 100.0 g of MMT was dispersed in deionized water in a 50 L reactor with mechanical agitation and high–low−temperature circulator at 85 °C, and the temperature of the reactor increased to 95 °C two hours later. Then, MCA hot water dispersion was slowly added to the reactor by a metering pump at a constant speed for about 30 min and the reaction continued to be stirred for 3 h. Finally, the product named 1# was obtained by centrifugation, drying and grinding.

(3) Mechanical intercalation

In total, 50.0 g MMT and 50.0 g MCA were ground and blended in a grinder at 10,000 rpm/min for 5 min, and the product named 0# was obtained.

### 2.3. Preparation of PA6/FR

PA6/FR was blended in the twin-screw extruder SHJ-20 according to Table 1. The temperature profile over the screw from the feeding to the die was 230 °C, 235 °C, 240 °C, 245 °C, 245 °C and 240 °C. After drying at 80 °C for 2 h, the extruded granules were molded by injection molding machine HTF80X1, and the temperature of the screw ranged from 245 °C to 230 °C.

### 2.4. Characterization

The FTIR spectra of MMT-MCA, MMT, MCA, CA and MA were recorded with a Nicolet 6700 Fourier transform infrared spectrometer for 32 scan times at a resolution of 4 cm^−1^. The transition mode was used and the wavenumber range was set from 4000 to 400 cm^−1^.

Scanning electron microscopy (SEM) was used to observe the microstructure of MCA, MMT and MMT-MCA with a Hitachi ultra-high resolution S-4800 scanning electron microscope. The accelerated voltage of image information acquisition is 3.0 kV. The accelerated voltage of collecting energy spectrum information (EDS) was 20.0 kV.

A MiNiFlex600 X-ray diffractometer (XRD) was used to characterize the crystal structure of MMT-MCA. The X-ray wavelength used for scanning was 1.54078 A, the scanning range was 2~50°, the step size was 0.02° and the scanning speed was 2°/min.

The morphologies of MMT-MCA were characterized with a transmission scanning electron microscope (TEM) (JEOL JEM-F200, Tokyo, Japan) with an accelerating voltage of 200 kV and an energy spectrum model of JED-2300T.

The thermal decomposition and thermal stability of PA6/AHP/MMT-MCA were measured with a 209 F1 Iris thermogravimetric analyzer (TGA). All samples were tested in a nitrogen atmosphere with a total flow rate of 50 mL /min, a heating rate of 10 °C/min and a temperature range of 40~800 °C.

**Figure 1:**
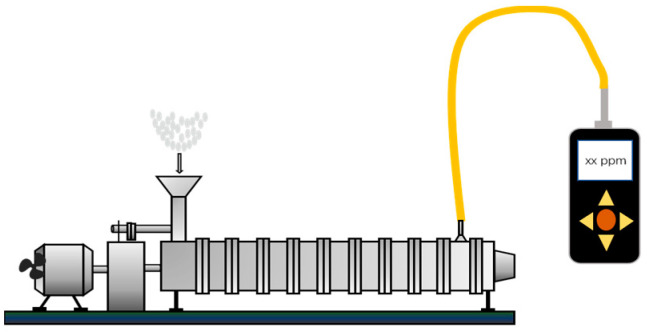
Illustration of real-time phosphine release concentration testing.

The real-time phosphine release concentration shown in Figure 1 was collected with a Plt400-PH_3_ phosphine detector with a range of 0–1000 ppm produced by Shenzhen Xinchuang Anda Electronic Technology Co., Ltd., Shenzhen, China, at the closest barrel to the die.

The TGA was coupled with a Fourier transform infrared spectrometer (TGA-FTIR, Nicolet 6700) to detect the PH_3_ gas release of flame retardant PA6 in sample 2#. TGA was performed with a Netzsch 209 F1 thermal analyzer, and the tests were carried out in the nitrogen atmosphere at a heating rate of 20 °C/min from 40 to 800 °C with a gas flow rate of 60 mL/min.

The UL-94 of PA6/FR was tested with a vertical combustion tester of (UL94-X) manufactured by Motis Combustion Technology (China) Co., LTD., Suzhou, China.

The LOI of PA6/FR was carried out with the intelligent critical oxygen index analyzer (TTECH-GBT2406-1) manufactured by Tectech (Suzhou) Testing Instrument Technology Co., LTD., Suzhou, China.

## 3. Results and Discussion

### 3.1. Structure of MMT-MCA

**Figure 2:**
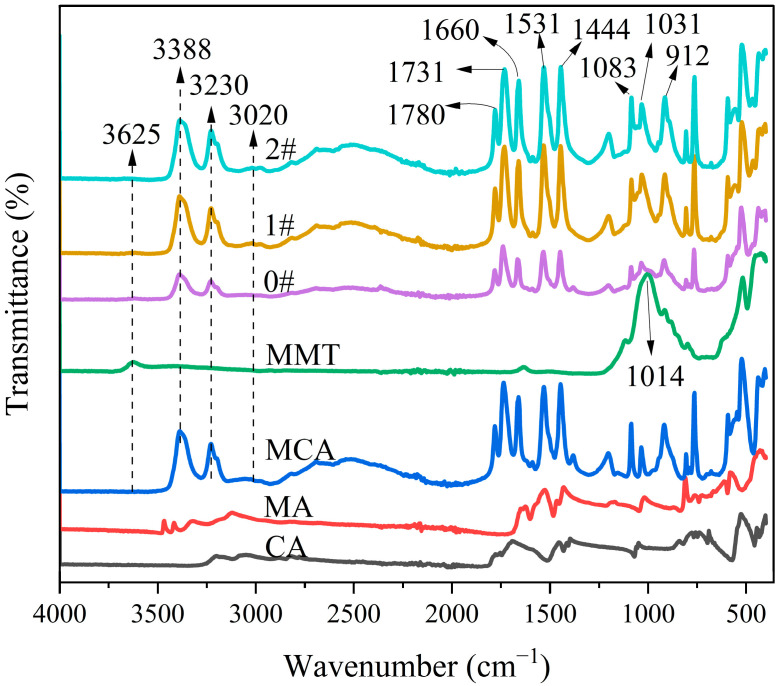
Characterization of FTIR spectra of CA, MA, MCA, MMT and modified MMT.

Figure 2 shows the FTIR spectra of CA, MA, MCA, MMT and MMT-MCA. The absorption at 3625 cm^−1^ was caused by the stretching vibration of the -OH group in the octahedral alumina sheet of MMT. The peak at 3388 cm^−1^ was caused by the symmetric stretching vibration of -NH_2_ of the triazine group [35]. The peaks at 3230 cm^−1^ and 3020 cm^−1^ were attributed to the formation of hydrogen bonds between the amino and imino groups [13]. The peak at 1780 cm^−1^ was attributed to the stretching vibration of the C=O of MCA. The strong peaks at 1731 cm^−1^ and 1660 cm^−1^ were caused by shear and bending vibrations of -NH_2_, respectively. The peaks at 1531 cm^−1^ and 1444 cm^−1^ were consistent with the stretching vibrations of C=N and C-N, respectively. The absorption peak at 1014 cm^−1^ was attributed to the bending vibrations of Si-O-Si and Al-O bonds, but this peak shape disappeared in the absorption pattern of 0#, 1# and 2# because the peak 1014 cm^−1^ was simply superimposed by the absorption peaks of 1083 cm^−1^, 1031 cm^−1^ and 912 cm^−1^ generated by MCA. The above results showed that MA and CA reacted successfully and MCA was synthesized in 2# [35,37], and the chemical structure of the three kinds of MMT-MCA did not change.

**Figure 3:**
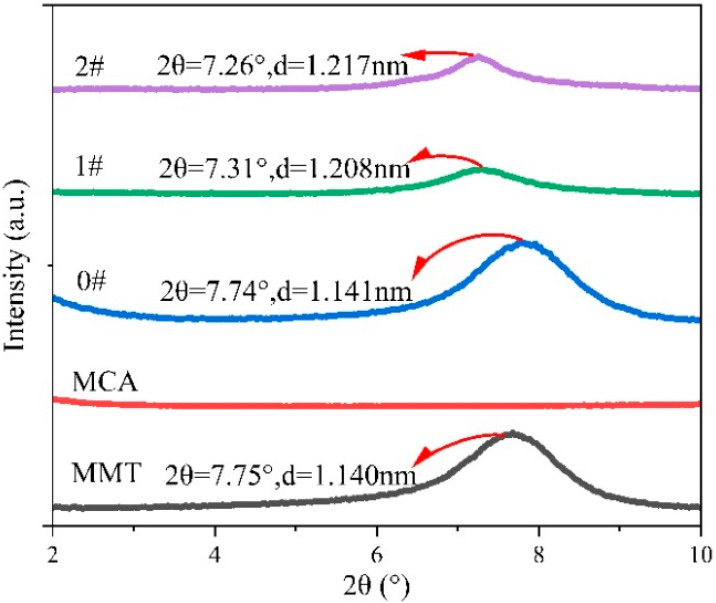
Characterization of XRD spectrum of MCA, MMT and MMT-MCA.

As shown in Figure 3, the crystal plane spacing of pure MMT was about 1.140 nm, and the spacing of MMT sheets in 0#, 1# and 2# was 1.141 nm, 1.208 nm and 1.217 nm, respectively. The slight increase in MMT sheet spacing of 0# may be attributed to the expansion of loose MMT sheets caused by mechanical fracturing. The MMT sheet spacing of 1# and 2# increased more obviously, contributing to the obvious expansion of MMT sheet spacing, and the effect of the in situ intercalation method on extending MMT sheet spacing was better.

### 3.2. Morphology of MMT-MCA

**Figure 4:**
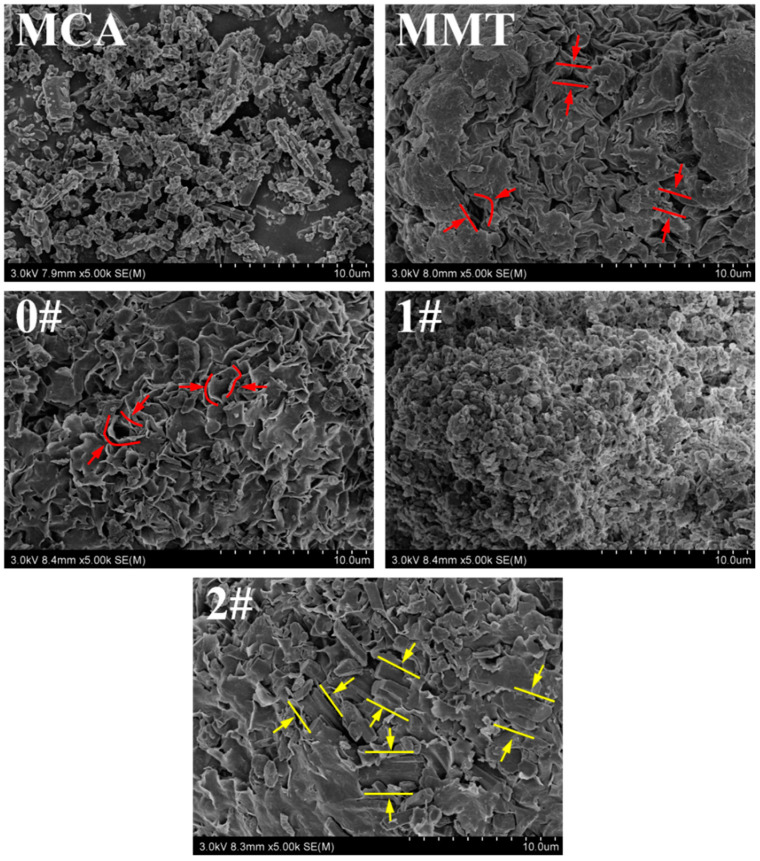
SEM images of MCA, MMT and MMT-MCA (0#, 1#, and 2#) at 3 kV accelerating voltage.

In Figure 4, MCA was a short rod-like crystal of MA and CA interleaved and hydrogen-bonded [38], while MMT was the product of the stacking of irregular layered structures with gaps matching MCA size between its sheets, shown as the red lines [25]. In 0#, MCA stick crystals were not observed between the sheets of MMT, which proved that the mechanical mixing without solvent could hardly implement the intercalation of MCA between the MMT sheets. In 1#, because of the hydrogen bonding between MCA and MMT, MCA was deposited on and covered the surface of MMT in large quantities, which was not conducive to the exfoliation of MMT [37]. However, in 2#, the MCA crystal grew inside the MMT and poked out between the MMT sheets, shown as yellow lines, which destroyed the layered stacking structure of the MMT and led to the partial exfoliation of the MMT sheets, promoting the exfoliation of the MMT. These phenomena were consistent with the shift of the 001 peak to a smaller angle in XRD [37], which further proved that MCA was successfully inserted into the MMT sheets and the in situ intercalation method had better intercalation efficiency than water intercalation.

**Figure 5:**
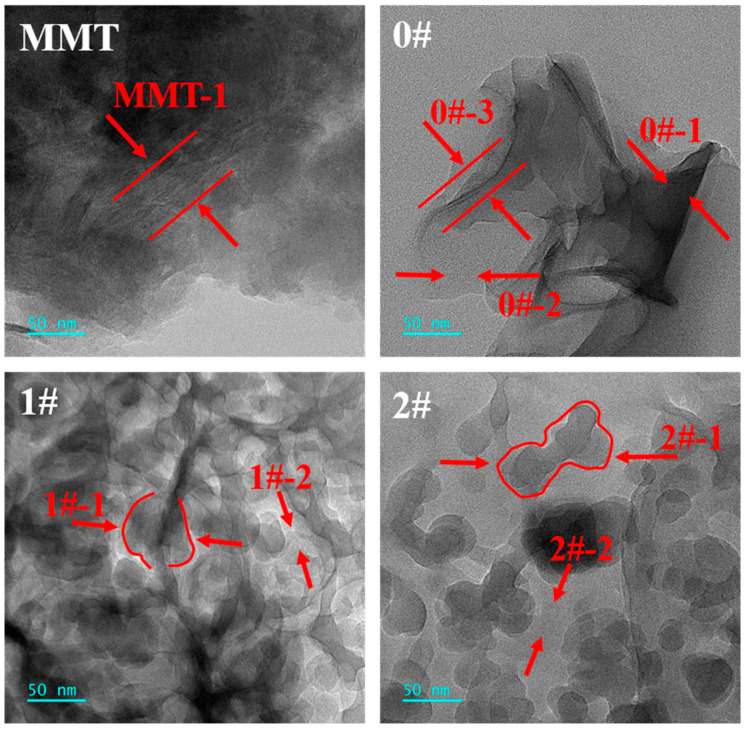
TEM images of MMT, MMT-MCA (0#, 1# and 2#) at 200 kV accelerating voltage.

Figure 5 shows the TEM images of MMT and MMT-MCA. Table 2 shows the element analysis of the corresponding position in the TEM images. Many filamentous structures can be clearly seen shown as the red arrows in MMT-1 and 0#-3 of Figure 5, which were caused by the sheets of MMT exactly parallel to the direction of the probe. In Figure 5, the cavity morphology of 1#-1 and 2#-1 shown as the arrows disappeared in MMT and 0#. According to the element analysis in Table 2, pure MMT contained almost no nitrogen element but carbon with 39.69 wt% content owing to the conductive carbon coating to facilitate TEM detection. Moreover, 0#-1 had significant content of nitrogen element, attributed to aggregation of MCA on the surface of MMT in mechanical mixing. However, 1#-1 and 2#-1 had significant content of not only the nitrogen element but also the cavity structure, because the barrier effect between layers of MMT prevented MCA from forming a continuous structure and TEM detective electrons could easily penetrate MCA based on hydrogen bonds [6]. This further confirmed that the water intercalation and in situ intercalation successfully inserted MCA into the sheets of MMT, while the mechanical intercalation could not achieve the intercalation of MCA into MMT, which was consistent with the results of XRD.

### 3.3. Thermal Stability of PA6/FR

**Figure 6:**
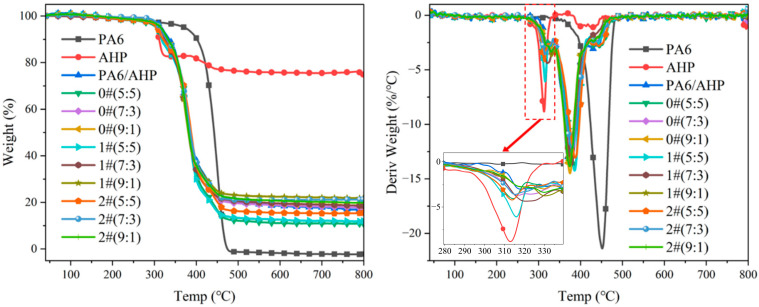
TGA-DTG curves of PA6/FR in nitrogen atmosphere.

TGA and DTG curves of pure PA6, PA6 loaded with 25 wt% AHP, and PA6 loaded with AHP modified with different proportions of MMT-MCA in nitrogen are shown in Figure 6, and the TGA data are listed in Table 3. The onset decomposition temperature (T_-5%_) obtained from Weight–Temp curves was considered as the temperature at the 5 wt% weight loss, and T_max_ obtained from Deriv Weight–Temp curves was defined as the temperature at the maximal mass loss rate. The experimental residual at 800 °C (ER) was given by TGA. The theoretical residual at 800 °C (TR) was estimated according to Equation (1), as follows:

TR = 6.75/25x + 0.7486x + y(1)

where x with unit “%” represents the mass content of AHP in PA6, and y with unit “%” represents the mass content of MMT in PA6. The gap ratio (GR) represents the gap between ER and TR, and is calculated by Equation (2):

GR = (TR − ER)/TR × 100%(2)

The onset degradation temperature (T_-5%_) was around 317 °C and T_max1_ was attributed to the degradation of the first degradation stage of AHP. T_max2_ was mainly related to the degradation of MCA and the acceleration of sublimation of melamine. T_max3_ was mainly related to the accelerated degradation of polyamide 6 and the decomposition of thermal decomposition products of AHP. With the increase in MMT-MCA, T_max1_ and the T_-5%_ of PA6/AHP loaded with MMT-MCA decreased, which was attributed to slight facilitatory effect of MCA on thermal degradation of AHP [13]. With the increase in MMT-MCA, compared with PA6/AHP, the T_max2_ and T_max3_ of PA6/AHP loaded with MMT-MCA rose, but the T_max2_ and T_max3_ only exceeded 380.2 °C with a high loading ratio of MMT-MCA, which was ascribed to the thermal protection of MMT [26]. The ER of PA6/AHP loaded with MMT-MCA decreased compared to that of PA6/AHP unloaded with MMT-MCA, attributed to the facilitatory effect of micromolecular degradation products of MCA on thermal degradation of PA6 [39,40,41]. However, the GR of 2#(7:3) was the minimum, because exfoliated MMT sheets had better thermal protection than untreated MMT but high loading could facilitate agglomeration of exfoliated MMT sheets [42]. These results indicated that 2# had better promotion than 0# and 1#, and exfoliated MMT sheets could reduce the promoting effect of MCA on the thermal degradation of PA6 and promote the char formation of PA6, and 2# had an optional loading mass ratio 3:7 with AHP.

### 3.4. LOI and UL 94 of PA6/FR

From Table 4, it can be seen that the LOI was the highest and the dripping of fire disappeared with PA6 loaded with 2# in the same mass ratio, owing to more efficient exfoliation of MMT sheets through in situ intercalation and the increase in the viscosity of the melted PA6 caused by exfoliated MMT sheets [25]. Although the LOI decreased with PA6 loaded with MMT-MCA, owing to the promoting effect of MCA on thermal degradation of PA6 [16], exfoliated MMT sheets formed a barrier to protect the matrix [25].

### 3.5. PH_3_ Release of PA6/FR

**Figure 7:**
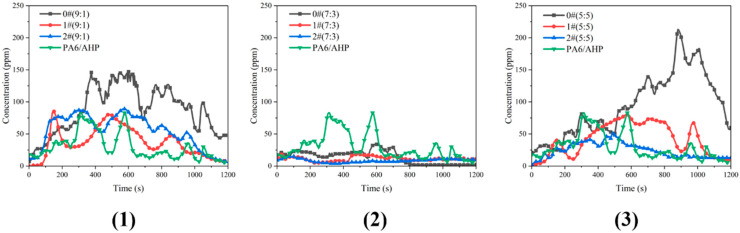
PH_3_ release curves of PA6/AHP modified at ratio of (**1**) 9:1, (**2**) 7:3 and (**3**) 5:5 in twin-screw processing.

Figure 7 shows the PH3 release curves during the twin-screw extrusion process. Table 5 shows the parameters from the PH3 release curves of PA6/FR in Figure 7. For all samples, the total weight of each group of samples was 1000 g according to the formulation in Table 1, and the extrusion was completed before 1000 s, and the gas collection time was 1200 s. As the ratio of AHP/MMT-MCA changed from 9:1 to 5:5, phosphine release decreased first and then increased, and PH3 release was significantly inhibited at 7:3. Moreover, the PA6 loaded with AHP modified with 1# or 2# showed better inhibition than that loaded with AHP modified with 0#, reflected by relatively low PH3 release curves. Furthermore, 2#(7:3) had the slowest release of PH3 throughout, and its peaks of PH3, mean values of PH_3_ and integral of PH_3_ were reduced by 81.9%, 72.1% and 72.2%, respectively. The results indicated that MMT-MCA from in situ intercalation was the best inhibitor of PH3 release.

**Figure 8:**
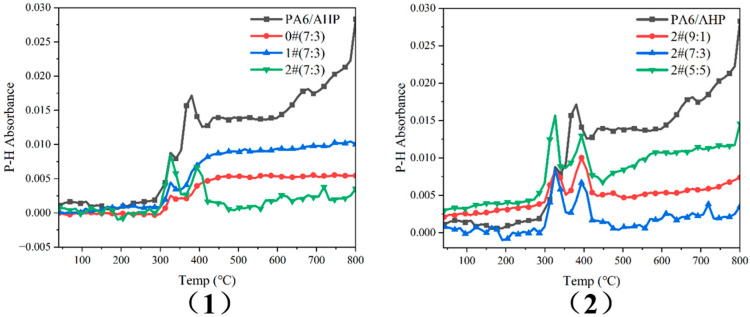
Infrared absorbance curves of P-H of (**1**) PA6/AHP loaded with 0#, 1# or 2# in the same mass ratio 7:3, (**2**) PA6/AHP loaded with 2# in the different mass ratio 9:1, 7:3 and 5:5, during the TGA testing.

TGA-FTIR was used to analyze the emission of PH_3_ gas of PA6/AHP treated by MMT-MCA during thermal decomposition, as shown in Figure 8. The infrared absorption intensity of the P-H bond in the gas phase reflected the concentration of phosphine gas. In Figure 8(1), it is clearly shown that MMT-MCA could reduce the release concentration of phosphine gas, and MMT-MCA from in situ intercalation could better inhibit the phosphine release than MMT-MCA from the other two methods. In Figure 8(2), the P-H absorption peak intensity of 2#(5:5) is larger than that of 2#(7:3), which clearly indicates that the loading of MMT-MCA 2# should not be blindly increased; otherwise, the agglomeration of MMT sheets may not be conducive to the adsorption and blocking of PH_3_ release.

### 3.6. MMT-MCA Distribution in PA6

**Figure 9:**
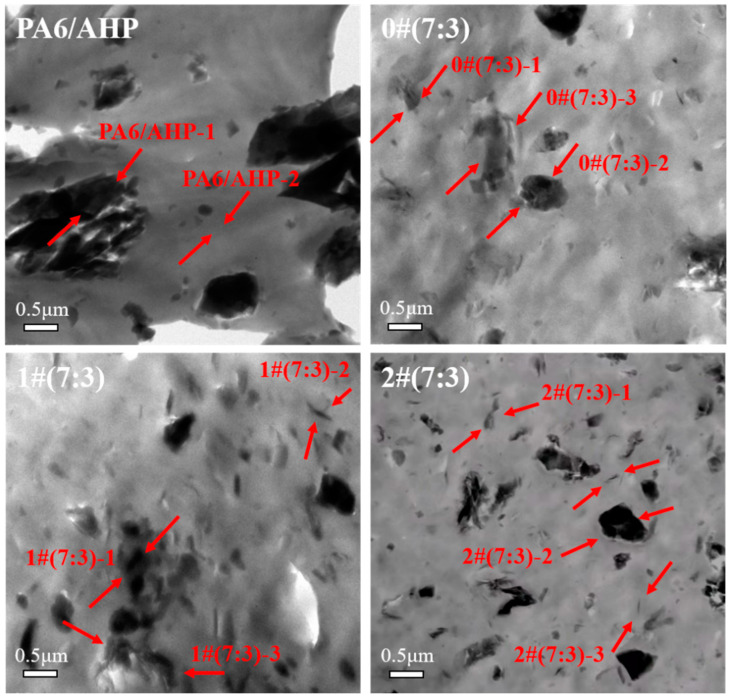
TEM images of PA6/AHP, 0#(7:3), 1#(7:3) and 2#(7:3) at 200 kV accelerating voltage.

Figure 9 shows that the distribution of additives in PA6 sliced at low temperatures that could be observed by the TEM. Table 6 shows the EDS labeled in Figure 9. AHP with irregular sizes were randomly distributed in PA6. A large number of two-dimensional sheet structures, which were proven to be MMT-MCA by TEM-EDS, as shown in Table 6, were observed in 1#(7:3) and 2#(7:3). However, few small two-dimensional sheet structures were observed in 0#(7:3), and many larger MMT particles were present. This showed that both water intercalation and in situ intercalation could promote the exfoliation of MMT when MMT-MCA and PA6 were processed in the twin-screw extruder. The adsorption of PH_3_ by MMT was significantly enhanced by two-dimensional modification [43,44]. Moreover, the uniformly distributed two-dimensional MMT sheets formed obstacles in the release path of PH_3_, so that the release of PH_3_ was slowed down, which was owing to the physical adsorption of MMT and the chemical bonding of MCA [13,19,20].

## 4. Conclusions

Montmorillonite–melamine cyanurate (MMT-MCA) could be prepared by both water intercalation and in situ intercalation, with the sheet spacing of MMT increasing from 1.140 nm to 1.208 nm and 1.217 nm, respectively, while mechanical intercalation made it hard to achieve MCA intercalation into MMT. Owing to in situ synthesis and diffusion, in situ intercalation was more conducive to the intercalation of MCA into MMT than water intercalation, and promotion of the distribution of MMT in the PA6 matrix. In addition, exfoliated MMT sheets could promote thermal stability, char formation and flame resistance of PA6.

The inhibition efficiency of MCA-MMT in terms of PH_3_ emission was related not only to the intercalation method but also to the critical addition amount of MMT-MCA. The optimal ratio of MMT-MCA to AHP was about 3:7, and in situ intercalation MMT-MCA had the highest inhibition efficiency in terms of PH_3_ release, because in situ intercalation had stronger exfoliation in terms of MMT in the PA6 matrix, and MMT of MMT-MCA from in situ intercalation with larger specific surface area could adsorb more PH_3_ molecules.

Overall, in situ intercalation MMT-MCA is a cheap, environmentally friendly and efficient way to promote the safety of AHP, which can promote the application of AHP.

## Figures and Tables

**Table 1:** Formula of PA6/FR.

Samples	PA6 (g)	AHP (g)	MMT-MCA (g)			Antioxid1010 (g)	Antioxid168 (g)	Calcium Stearate (g)
			0#	1#	2#			
PA6	792	0	0	0	0	1	2	5
PA6/AHP	742	250	0	0	0	1	2	5
0#(9:1)	742	225	25	0	0	1	2	5
0#(7:3)	742	175	75	0	0	1	2	5
0#(5:5)	742	125	125	0	0	1	2	5
1#(9:1)	742	225	0	25	0	1	2	5
1#(7:3)	742	175	0	75	0	1	2	5
1#(5:5)	742	125	0	125	0	1	2	5
2#(9:1)	742	225	0	0	25	1	2	5
2#(7:3)	742	175	0	0	75	1	2	5
2#(5:5)	742	125	0	0	125	1	2	5

**Table 2:** The contents of elements C, N and Si of the marked positions in Figure 5 through EDS.

Positions	C (wt%)	N (wt%)	Si (wt%)
MMT-1	39.69	1.19	59.12
0#-1	59.13	36.90	3.96
0#-2	61.92	3.31	34.77
1#-1	63.40	27.47	9.13
1#-2	62.18	19.66	18.16
2#-1	56.86	26.10	17.04
2#-2	57.77	16.78	25.45

**Table 3:** TGA-DTG data of TGA of PA6/FR.

Samples	T_-5%_ (°C)	T_max1_ (°C)	T_max2_ (°C)	T_max3_ (°C)	TR at 800 °C (%)	ER at 800 °C (%)	GR (%)
PA6	372.6	\	\	451.9	\	0	\
AHP	303.5	312.9	\	434.0	\	74.86	\
PA6/AHP	317.4	319.8	380.2	437.0	25.47	25.47	0
0#(5:5)	304.4	314.4	386.5	442.4	18.98	10.98	42.2
0#(7:3)	311.6	316.5	379.5	436.1	21.58	17.70	18.0
0#(9:1)	312.5	321.5	374.9	432.7	24.17	18.74	22.5
1#(5:5)	306.3	315.8	385.7	441.3	18.98	11.91	37.2
1#(7:3)	312.8	320.5	380.6	433.1	21.58	18.58	13.9
1#(9:1)	313.8	329.8	372.4	432.0	24.17	21.84	9.6
2#(5:5)	309.2	314.9	381.7	445.3	18.98	11.15	41.2
2#(7:3)	314.0	315.3	380.0	441.0	21.58	21.24	1.6
2#(9:1)	314.8	331.2	378.3	433.4	24.17	23.32	3.5

**Table 4:** The results of LOI and UL 94 of PA6/FR.

Samples	UL-94		LOI (%)
	Dripping	Grade	
PA6	Yes	None	20%
PA6/AHP	No	V-0	26.0
0#(5:5)	Yes	None	24.5
0#(7:3)	No	V-0	24.8
0#(9:1)	No	V-0	25.2
1#(5:5)	Yes	None	24.7
1#(7:3)	No	V-0	25.5
1#(9:1)	No	V-0	25.3
2#(5:5)	No	V-0	25.0
2#(7:3)	No	V-0	25.8
2#(9:1)	No	V-0	25.7

**Table 5:** Parameters from PH_3_ release curves of PA6/FR.

Samples	Peaks of PH_3_ (ppm)	Means of PH_3_ (ppm)	Integral of PH_3_ (ppm·s)
PA6/AHP	83	29.8	35,830
0#(9:1)	147	86.8	104,147
0#(7:3)	34	13.9	16,673
0#(5:5)	213	94.5	113,468
1#(9:1)	86	37.0	44,410
1#(7:3)	19	11.4	13,694
1#(5:5)	80	42.0	50,385
2#(9:1)	89	54.4	65,345
2#(7:3)	15	8.3	9977
2#(5:5)	53	24.4	29,244

**Table 6:** TEM-EDS of labeled positions in Figure 9.

Positions	C (wt%)	N (wt%)	Al (wt%)	Si (wt%)	P (wt%)
PA6/AHP-1	10.95	65.85	6.58	0.04	16.58
PA6/AHP-2	53.93	45.95	0.04	0.05	0.03
0#(7:3)-1	15.65	66.56	3.43	14.21	0.14
0#(7:3)-2	18.01	37.63	7.18	0.42	36.77
0#(7:3)-3	79.00	3.02	5.64	10.32	2.02
1#(7:3)-1	11.99	65.95	6.14	0.9	15.02
1#(7:3)-2	33.90	56.28	3.41	6.17	0.19
1#(7:3)-3	23.53	65.07	1.50	9.85	0.06
2#(7:3)-1	29.57	60.11	3.89	6.15	0.28
2#(7:3)-2	52.80	38.40	2.77	0.09	5.94
2#(7:3)-3	43.33	56.35	0.02	0.38	0.02

## Data Availability

The raw/processed data required to reproduce these findings cannot be shared at this time due to technical or time limitations.
